# A Genome Scan for Genes Underlying Microgeographic-Scale Local Adaptation in a Wild *Arabidopsis* Species

**DOI:** 10.1371/journal.pgen.1005361

**Published:** 2015-07-14

**Authors:** Shosei Kubota, Takaya Iwasaki, Kousuke Hanada, Atsushi J. Nagano, Asao Fujiyama, Atsushi Toyoda, Sumio Sugano, Yutaka Suzuki, Kouki Hikosaka, Motomi Ito, Shin-Ichi Morinaga

**Affiliations:** 1 Graduate School of Arts and Sciences, The University of Tokyo, Meguro, Tokyo, Japan; 2 College of Bioresource Sciences, Nihon University, Fujisawa, Kanagawa, Japan; 3 Center for Sustainable Resource Science, RIKEN, Yokohama, Kanagawa, Japan; 4 Frontier Research Academy for Young Researchers, Kyushu Institute of Technology, Iizuka, Fukuoka, Japan; 5 CREST, Japan Science and Technology Agency, Kawaguchi, Saitama, Japan; 6 Center for Ecological Research, Kyoto University, Otsu, Shiga, Japan; 7 PRESTO, Japan Science and Technology Agency, Kawaguchi, Saitama, Japan; 8 Center for Information Biology, National Institute of Genetics, Mishima, Shizuoka, Japan; 9 Graduate School of Frontier Sciences, The University of Tokyo, Kashiwa, Chiba, Japan; 10 Graduate School of Life Sciences, Tohoku University, Sendai, Miyagai, Japan; University of Georgia, UNITED STATES

## Abstract

Adaptive divergence at the microgeographic scale has been generally disregarded because high gene flow is expected to disrupt local adaptation. Yet, growing number of studies reporting adaptive divergence at a small spatial scale highlight the importance of this process in evolutionary biology. To investigate the genetic basis of microgeographic local adaptation, we conducted a genome-wide scan among sets of continuously distributed populations of *Arabidopsis halleri* subsp. *gemmifera* that show altitudinal phenotypic divergence despite gene flow. Genomic comparisons were independently conducted in two distinct mountains where similar highland ecotypes are observed, presumably as a result of convergent evolution. Here, we established a *de novo* reference genome and employed an individual-based resequencing for a total of 56 individuals. Among 527,225 reliable SNP loci, we focused on those showing a unidirectional allele frequency shift across altitudes. Statistical tests on the screened genes showed that our microgeographic population genomic approach successfully retrieve genes with functional annotations that are in line with the known phenotypic and environmental differences between altitudes. Furthermore, comparison between the two distinct mountains enabled us to screen out those genes that are neutral or adaptive only in either mountain, and identify the genes involved in the convergent evolution. Our study demonstrates that the genomic comparison among a set of genetically connected populations, instead of the commonly-performed comparison between two isolated populations, can also offer an effective screening for the genetic basis of local adaptation.

## Author Summary

Where does a local adaptation take place? In general, an adaptive divergence is predicted to occur between isolated populations because gene flow will erode and prevent the divergence. Therefore, previous genome-wide studies that aim to find the adaptive genes have compared populations that are usually tens of hundreds of kilometers apart. However, because nearby populations are likely to be genetically connected or connected until recently, most of the genome should be undifferentiated, leaving the genetic footprints of natural selections more pronounced. Thus, if an adaptive divergence is to be found within a small spatial scale, such case may favor the screening for the adaptive genes. Here, we took advantage of a unique small-scale local adaptation in *Arabidopsis halleri* subsp. *gemmifera*, where similar phenotypic differentiation is found across an altitudinal cline on two distinct mountains. By scanning the genome with a focus on the presence of unidirectional allele frequency shift along the altitudes, we successfully obtained genes with functions that were in line with the known phenotypic and environmental difference between altitudes. Our approach is applicable to any species that show microgeographic divergence and should help understand the genetic basis of small-scale evolution.

## Introduction

Recent advances in next-generation sequencing (NGS) technologies have enabled a genome-scale analysis to infer the phylogenetic history, demography, and selection of natural populations. One of the intriguing challenges in ecological genomics is to identify the genes underlying local adaptation [1]. Although ecological genomics has been applied to various study systems, screening methods to detect the selected loci can be represented by two approaches: those that focus on the adaptive differentiation, and those that focus on the genotype-environment correlations. The former differentiation-based approach assumes neutral genetic drift to affect the entire genome, so that unusual differentiation at a particular locus should indicate a presence of selection. *F*_*ST*_-based outlier tests are among the earliest and most common method to detect the selected loci [2]. The latter correlation-based approach compares a set of subpopulations at heterogeneous environments to detect the loci with correlation between allele frequency and environmental variables [3]. Availability of the genome-scale datasets have facilitated improvements in these two approaches, along with the development of other methods that employ indicators such as reduced heterozygosity, skews in site frequency spectrum, and extended linkage disequilibrium (reviewed in [4]). Although ecological genomics have provided important insights into the genetic basis of local adaptation, each of the above mentioned approaches has drawbacks to its practical implementation, which includes false positive and false negative detection of the selected loci. For instance, *F*_*ST*_-based outlier tests generally face problems in identifying the significant departure from neutral expectation. Without taking account the actual demographic history, outlier tests may suffer from false positives due to high variance in *F*_*ST*_ values among the neutral loci [5]. Within- and between-population structures can also increase the false positive rate of correlation-based approaches by creating spurious correlation between allele frequency and environmental variable [6]. In any case, complex demographic histories and entailing genetic structures are the major issues that challenge the genome-wide screening for adaptive genes, and a combination of different approaches is preferred to avoid false detections [6].

Because gene flow will erode and prevent a genetic divergence, adaptive differentiation is more likely to occur between populations that are reproductively isolated. Geographical distance can provide a strong reproductive barrier and also shape environmental differences (e.g., temperature along the latitudes), both of which may facilitate the adaptive divergence between populations. Indeed, most ecological genomic studies compare populations that are tens of hundreds of kilometers apart (e.g. representative study cases reviewed in [7]). The problem of comparing distantly isolated populations is that the periods since population divergence are usually long enough to allow the intervention of various demographic processes. As a consequence, complicated population structure seems as an intrinsic difficulty to conduct the genome-wide scan for adaptive genes. Recently, growing number of works reporting microgeographic-scale adaptation [8–12] have corroborated the theory that adaptive population divergence can take place even under high gene flow if selective pressure is sufficient [13]. Microgeographic-scale adaptation may in fact be a suitable system for ecological genomics because the evolutionary split between nearby populations should be relatively recent compared to that of distantly isolated populations. Furthermore, gene flow may benefit the screening procedure because most of the genome is expected to be undifferentiated between populations, leaving the genetic footprints of a natural selection more pronounced [14]. In plant species, NGS-based restriction-site associated DNA (RAD) sequencing has been used to study the distinct ecotypes that occur within few kilometers from each other in *Senecio* [15], and *Helianthus* [16]. Although these studies have provided insights into the phylogenetic history, population demography, and genomic structure dynamics during microgeographic-scale divergence, candidate genes that underlie the phenotypic differentiation were not identified.

An example of microgeographic-scale divergence has been reported from a self-incompatible perennial plant, *Arabidopsis halleri* subsp. *gemmifera*. In Mt. Ibuki, a mountain located in central Japan, populations of this plant are continuously distributed along the top to bottom of a hiking trail. Although the linear distance between the lowest and highest populations is smaller than 3 km, highland ecotypes characterized by dense trichomes on the leaves and stems [17] are found on the peaks (S1 Fig). A previous AFLP-based study on Mt. Ibuki demonstrated little genetic differentiation between normal and highland ecotypes collected from low and high altitudes [18]. Thus, it has been suggested that these two ecotypes share a similar genomic structure and the evolutionary split has occurred relatively recently. Interestingly, similar phenotypic divergence is also found along the altitudes of Mt. Fujiwara, which situate approximately 30 km from Mt. Ibuki. Highland ecotypes of the two mountains are regarded as a convergent evolution, however, no empirical evidences have yet been reported. In addition to denser trichomes, growth chamber measurements have confirmed other genetically based convergent characteristics of the highland ecotypes, such as shorter but thicker stems and leaves, increased resource investment to photosynthetic components, and increased accumulation of ultraviolet (UV) absorbing compounds [19]. Overall, these altitudinal differentiations are considered as a consequence of high altitude adaptation. Although trichomes in plants often serve in the defense against herbivores [20], a study in *A*. *halleri* subsp. *gemmifera* revealed no clear correlation with leaf beetle damage [21]. Interestingly, the hyperaccumulator plant *A*. *halleri* accumulates zinc and cadmium inside its trichome bases [22]. This finding suggests that denser trichomes in the highland ecotypes might indicate higher tolerance to heavy metals. Alternative trichome functions in other plant species, including the prevention of external ice formation [23], avoidance of excess transpiration under strong wind [24], and protection against UV radiation [25], are also considered to be related to the adaptive significance of dense trichomes at high altitudes. Other characteristics of the highland ecotypes are also associated with the common selective pressures in the two mountains, such as dwarf phenotype to resist strong wind, investment to photosynthetic component to compensate the reduced enzyme activity due to suboptimal conditions, and accumulation of UV absorbing compound to tolerate increased UV radiation [19]. However, mountain-specific altitudinal differentiations are also reported. For instance, freezing resistance [19] and rapid seed germination (Shin-Ichi Morinaga, personal communications) are found only in the highland ecotypes from Mt. Ibuki. Nevertheless, the two mountains share similar environmental characteristics in terms of altitudinal cline. Although both mountains are relatively low (1,377 and 1,144 m for Mt. Ibuki and Mt. Fujiwara, respectively), areas above approximately 1,000 m are host to open subalpine grasslands with calcareous scree and heavy snow cover in winter. In contrast, areas lower than approximately 400m occupy the understory of temperate forests. Annual temperature, snow depth, and canopy openness have been quantified to show gradient variation along the altitude in both mountains [19]. As in this case, mountain populations may be an excellent model for the analysis of microgeographic adaptation because steep environmental gradients can shape selective barriers on a small geographic scale.

Thanks to the genetic information accumulated in *A*. *thaliana*, ecological genomics has become a powerful approach to screen adaptive genes from wild *Arabidopsis* species [26–29]. However, while these studies have provided fruitful insights into the genetic basis of local adaptations, genomic comparisons have so far been conducted at the macrogeographic-scale, using distantly isolated populations. Here, we test the prediction that genomic comparison at the microgeographic-scale can also offer an effective screening for the genetic basis of local adaptation. If the screening procedure works as expected, we should be able to find some correlation between the candidate genes and the observable phenotypic or environmental differentiation. In addition, a replicated analysis in two independent but synchronizing environmental transects will have a good chance of finding the genes involved in a convergent evolution. Our study system take advantage of the above mentioned populations of *A*. *halleri* subsp. *gemmifera* on Mt. Ibuki and Mt. Fujiwara, where populations continuously distribute along a steep environmental cline and the populations at each extreme (the lowest and highest populations on each mountain) are locally adapted to their habitats. Within each mountain, the loci governing altitudinal adaptation should be highly differentiated between the lowest and highest populations. More importantly, theoretical models predict that, if a set of populations is distributed along an environmental continuum and neighboring populations are exchange their genes, clines of allele frequencies at the adaptive loci can be observed [30, 31]. Because neighboring populations of *A*. *halleri* subsp. *gemmifera* in both mountains are close enough to allow gene flow, we placed an emphasis on detecting correlations between allele frequencies and altitudinal clines. Thus, we employed both differentiation-based and correlation-based approaches to screen the selected loci from a genome-wide SNP dataset. Credibility of the screening procedure was evaluated by comparing the proportion of a certain Gene Ontology (GO) term between screened and unscreened set of genes. Here, we selected 30 GO terms that cover the representative phenotypic and environmental entries within the database. If we successfully retrieve the genes under natural selection, then we should be able to see coincidence between the enriched GO terms and the known phenotypic or environmental differentiation across the altitudes. Furthermore, the screened loci were narrowed based on the presence of genetic hitchhiking. The screening procedure was independently applied to each mountain, and we obtained two lists of candidate genes that are potentially involved in altitudinal adaptation. By comparing these gene lists, we distinguished between genes that are adaptive only in either mountain, and those involved in the convergent evolution.

## Results and Discussion

### Establishment of the draft reference genome and the genome-wide SNP dataset

To perform a genome-wide screen for loci associated with local altitudinal adaptation, we began by establishing a draft *de novo* reference genome for *A*. *halleri* subsp. *gemmifera*. The whole-genome shotgun method via next-generation sequencing (NGS) was applied to a single individual sampled from the base of Mt. Ibuki. Using 190× coverage sequence data (haploid genome size of *A*. *halleri* = 255 Mbp [32]), genome assembly resulted in 149,013 scaffolds, with an N50 of 4,825 bp and a total of 252 Mbp, which corresponds to 98.8% of the entire genome. The resulting reference genome was evaluated by mapping *A*. *thaliana* exon sequences from 33,602 genes deposited in the TAIR10 database (The Arabidopsis Information Resource; http://www.arabidopsis.org). For comparison, we mapped the same *A*. *thaliana* exon sequences to the high-quality reference genome of *A*. *lyrata* (695 scaffolds, with an N50 of 24.5 Mbp, totaling 207 Mbp [33]). As a result, 92.9% and 90.7% of the *A*. *thaliana* exons were mapped to the reference genomes of *A*. *halleri* subsp. *gemmifera* and *A*. *lyrata*, respectively. Although the number of scaffolds remains excessive compared with the actual chromosome number in *A*. *halleri* (2n = 16; [32]), our draft *de novo* reference genome sequence covers the entire genome well and will facilitate genomic studies in this species.

**Fig 1:**
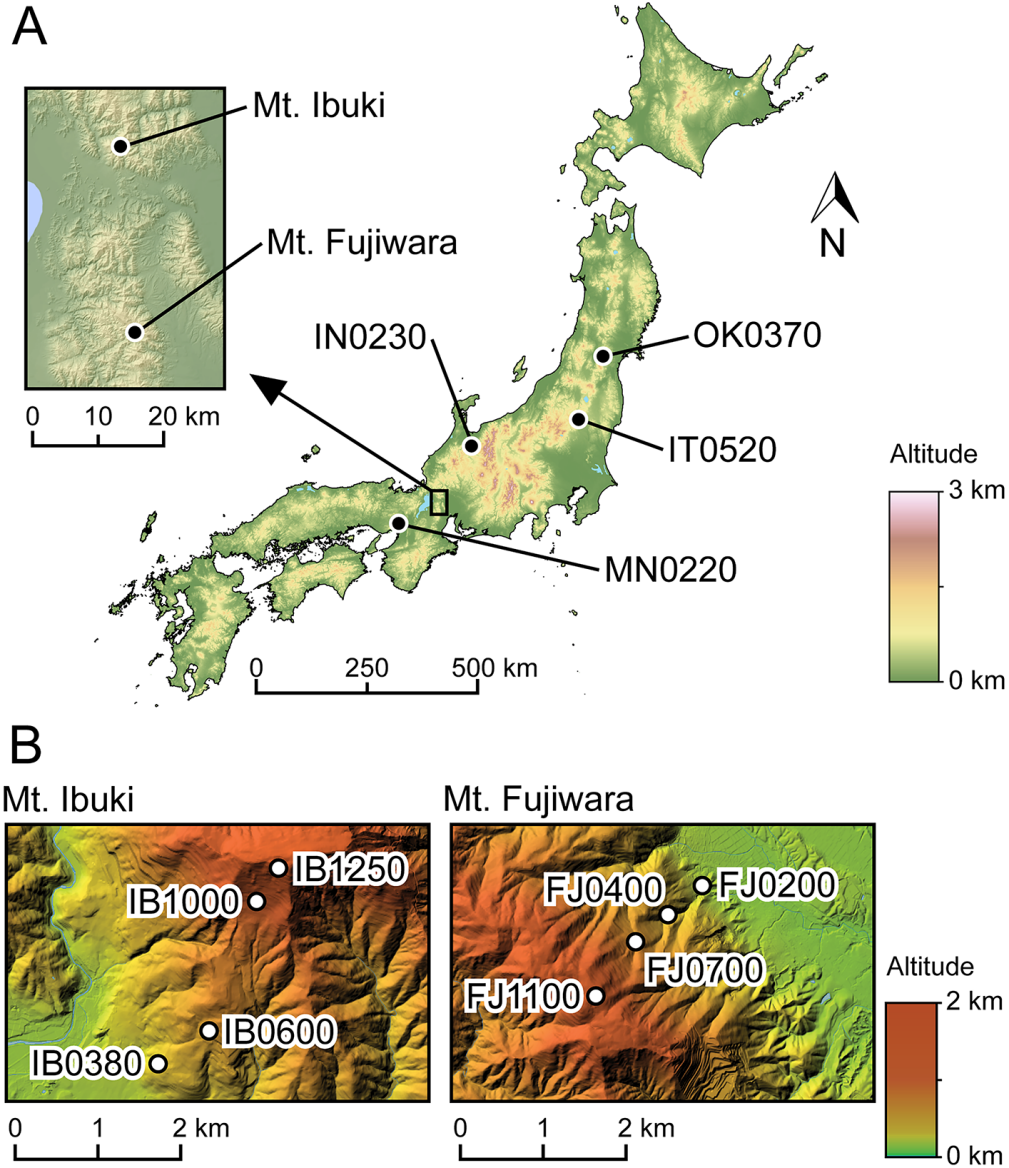
Location and of the studied populations. (A) Geographic locations of the two mountains (the main study sites) and the four low-altitude reference populations. Altitude is indicated by the numbers in the population names. See S1 Table for coordinates. (B) Locations of the four altitude-specific populations on each mountain.

On both Mt. Ibuki and Mt. Fujiwara, four distinct populations associated with different altitudes were situated along hiking trails from the bottom to the top of the mountains. The four populations are found at the altitudes of 380, 600, 1,000, and 1,250 m on Mt. Ibuki and at 200, 400, 700, and 1,100 m on Mt. Fujiwara (Fig 1B and S1 Table). The linear distance between the lowest and highest populations is approximately 2.7 km on Mt. Ibuki and 1.9 km on Mt. Fujiwara. In addition to the main study sites, four reference populations were set apart from the mountains (Fig 1A and S1 Table). These populations were situated at low altitudes (220, 230, 370, and 520 m) with environments similar to the lowest populations from the main study sites. On the two mountains, five individuals from each altitude-specific population were collected for analysis, whereas four individuals were collected from the reference populations. Through genome-wide resequencing of each of these 56 individuals, we obtained a set of 527,225 reliable SNPs with a minimum read count of five per individual (S1 Table). The average inter-SNP spacing across the entire genome was 484 bp. The mapped *A*. *thaliana* exon information was used to examine the proximity of each SNP to a functional gene. Among the 527,225 SNPs, 327,980 overlapped with or were within 5 kbp of an exon for 22,395 genes. These SNPs and the associated functional gene information were used for the following analyses.

### Genetic structure of the populations

**Fig 2:**
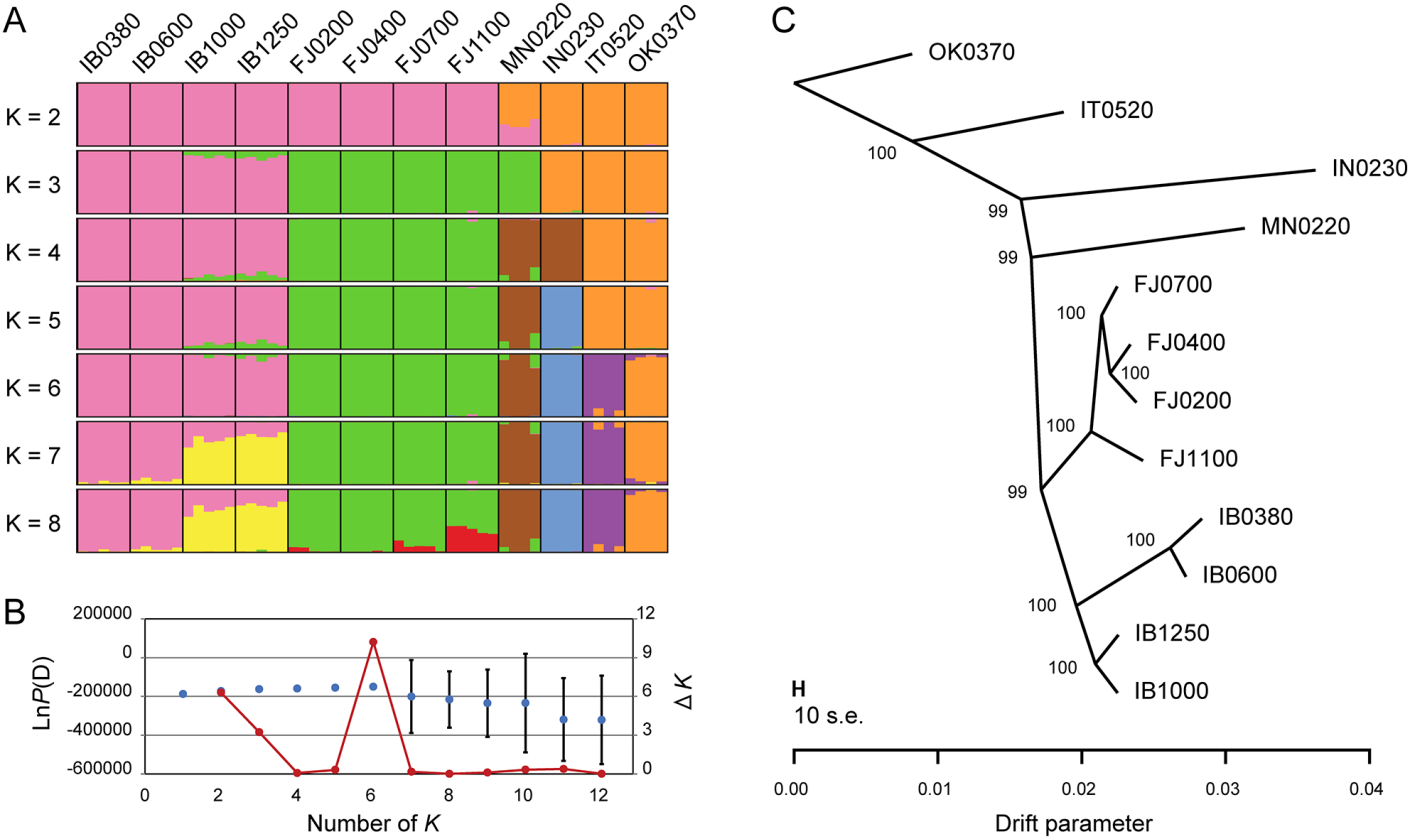
Genetic structures of the populations. (A) structure analysis with a *K* of 2 to 8 using all 56 individuals from the 12 populations. The result for each *K* is based on the simulation that provided the best Ln*P*(D) value (the log probability value) among 20 independent runs. Each bar represents an individual and the estimated membership in a particular genetic cluster. (B) Plotting of the mean Ln*P*(D) values from the structure analysis (blue dots) and Evanno’s Δ*K* (red dots). Error bars indicate the standard deviation of Ln*P*(D) values from the 20 independent runs. Both the maximum value of Ln*P*(D) and the peak position of Evanno’s Δ*K* are found at *K* = 6. (C) Maximum likelihood tree for the 12 populations obtained from TreeMix. The bootstrap supports for the nodes were calculated from 100 replicates. The scale bar represents 10 times the average standard error of the entries in the covariance matrix. Horizontal branch lengths are proportional to the amount of genetic drift. See S2 Fig for additional analysis within each mountain.

Genetic diversity (*H*_*e*_) was significantly different (bonferroni-corrected *p*-value from pairwise Wilcoxon test < 0.01) among all paired populations within each mountain, except for IB0380 vs. IB0600 in Mt. Ibuki, and FJ0400 vs. FJ1100 in Mt. Fujiwara (Table 1). Although the statistical significance is somewhat overestimated, lower populations of Mt. Ibuki tended to have smaller genetic diversity compared to higher populations. To examine the population structure within and between the two mountains, we conducted a structure [34, 35] analysis of all 56 individuals (including the reference populations) using a set of 10,000 randomly selected SNPs. Based on 20 independent runs for each value of *K* (the number of subpopulations) from 1 to 12, both the log likelihood value and Evanno’s Δ*K* method [36] indicated the optimum *K* to be six (Fig 2B). Under *K* = 6, each cluster clearly corresponded to the two mountains and the four reference populations (Fig 2A). It is notable that the four altitude-specific populations on each mountain were not genetically subdivided. However, subdivision within each mountain were indicated with higher *K* values. Further structure analysis within each mountain supported the split in Mt. Ibuki, but not in Mt. Fujiwara (S2 Fig). Previous study has demonstrated that although snow depth and canopy openness increased with increasing altitude in both mountain, Mt. Ibuki showed steeper gradients for both environmental components [19]. Thus, the genetic split in Mt. Ibuki may indicate a restricted gene flow among the altitudes due to stronger environmental barrier. Nevertheless, interleaving populations of Mt. Ibuki (IB0600 and IB1000) seem to be comprised of some admixed individuals. These individuals indicate the presence of gene flow between the neighboring altitude-specific populations. In fact, although highland ecotypes from the top of the mountain are easily distinguished based on their appearance, plants with intermediate phenotypes are found at intervening altitudes. Because highland and normal ecotypes are highly cross-compatible (Shin-Ichi Morinaga, personal communications), these intermediate plants are likely to have originated from natural hybridization due to frequent gene flow between neighboring populations. In addition, pairwise *G*′_*ST*_ values showed a pattern of genetic differentiation by distance in both mountains (Table 1). Thus, the population structure in each mountain can be regarded as a simple linear stepping-stone model proposed by Kimura and Weiss (1964 [37]).

**Table 1:** Summary statistics of genetic diversity and differentiation among the altitude-specific populations.

		Heterozygostiy statistics		Pairwise *G*'_ST_		
**Mt. Ibuki**	***P***	***H***_**e**_	***H***_**o**_	**vs. IB0380**	**vs. IB0600**	**vs. IB1000**
IB0380	0.162	0.051	0.047			
IB0600	0.162	0.050	0.046	0.027		
IB1000	0.224	0.064	0.053	0.046	0.043	
IB1250	0.209	0.061	0.056	0.048	0.046	0.034
**Mt. Fujiwara**	***P***	***H***_**e**_	***H***_**o**_	**vs. FJ0200**	**vs. FJ0400**	**vs. FJ0700**
FJ0200	0.224	0.066	0.055			
FJ0400	0.204	0.060	0.049	0.034		
FJ0700	0.228	0.064	0.056	0.036	0.034	
FJ1100	0.196	0.061	0.042	0.043	0.041	0.037

*P*, proportion of polymorphic loci

*H*_e_, mean expected heterozygosity

*H*_o_, mean observed heterozygosity.

We also examined the historical relationship among populations with TreeMix [38], a statistical model used to infer patterns of population splits and mixtures from genome-wide allele frequency data. The maximum likelihood tree based on 518,706 bi-allelic SNPs clearly demonstrated that the evolutionary split between the two mountains predated the differentiation of the altitude-specific populations (Fig 2C). In addition, the tree explained most (99.1%) of the variance in relatedness between the populations, which indicates that the tree captures the historical relationship without adopting migration events from distantly related populations. These results indicate that although the two mountains share a common ancestry, the differentiation of the altitude-specific populations took place independently on each mountain. Therefore, the morphologically similar highland ecotypes found on the two mountains may be considered to be a consequence of convergent evolution. Together with the results from structure analysis, these findings suggest that these populations are a suitable model for exploration of the genetic basis of microgeographic adaptation.

### Screening for SNPs associated with altitudinal adaptation

**Fig 3:**
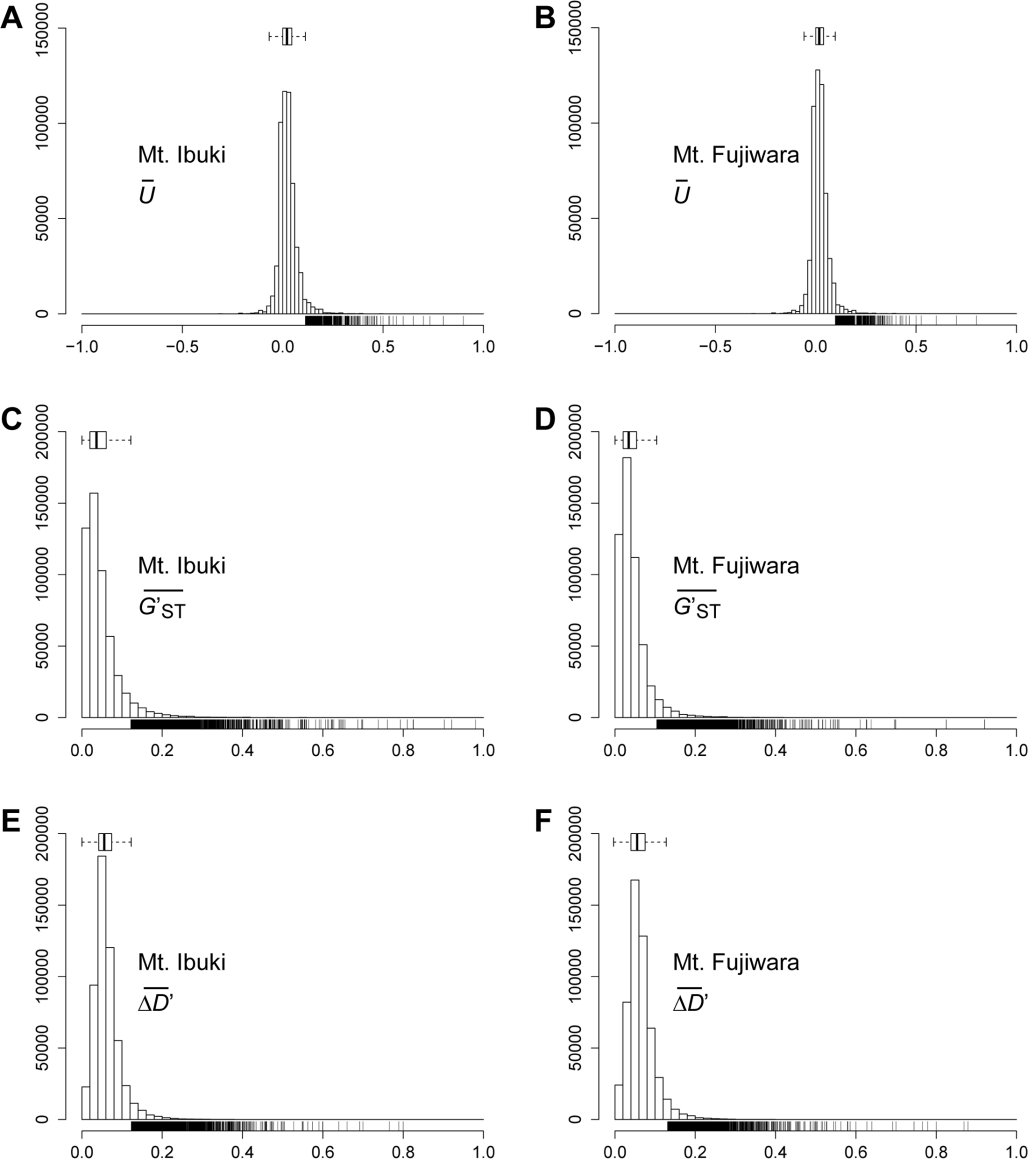
Genome-wide frequency distribution of the three indices. Histograms show the frequency distribution of $\bar{U}$ (A, B), $\bar{G\prime_{ST}}$ (C, D), and $\bar{\Delta D\prime }$ (E, F) estimated for all 527,225 SNPs in Mt. Ibuki (A, C, E) and Mt. Fujiwara (B, D, F). Box plots for each index are shown above the histogram. Whiskers of the box plot indicates the 1.5 times the IQR (interquartile range). Spikes below the histograms show the SNPs that fulfilled the threshold for each criterion.

**Fig 4:**
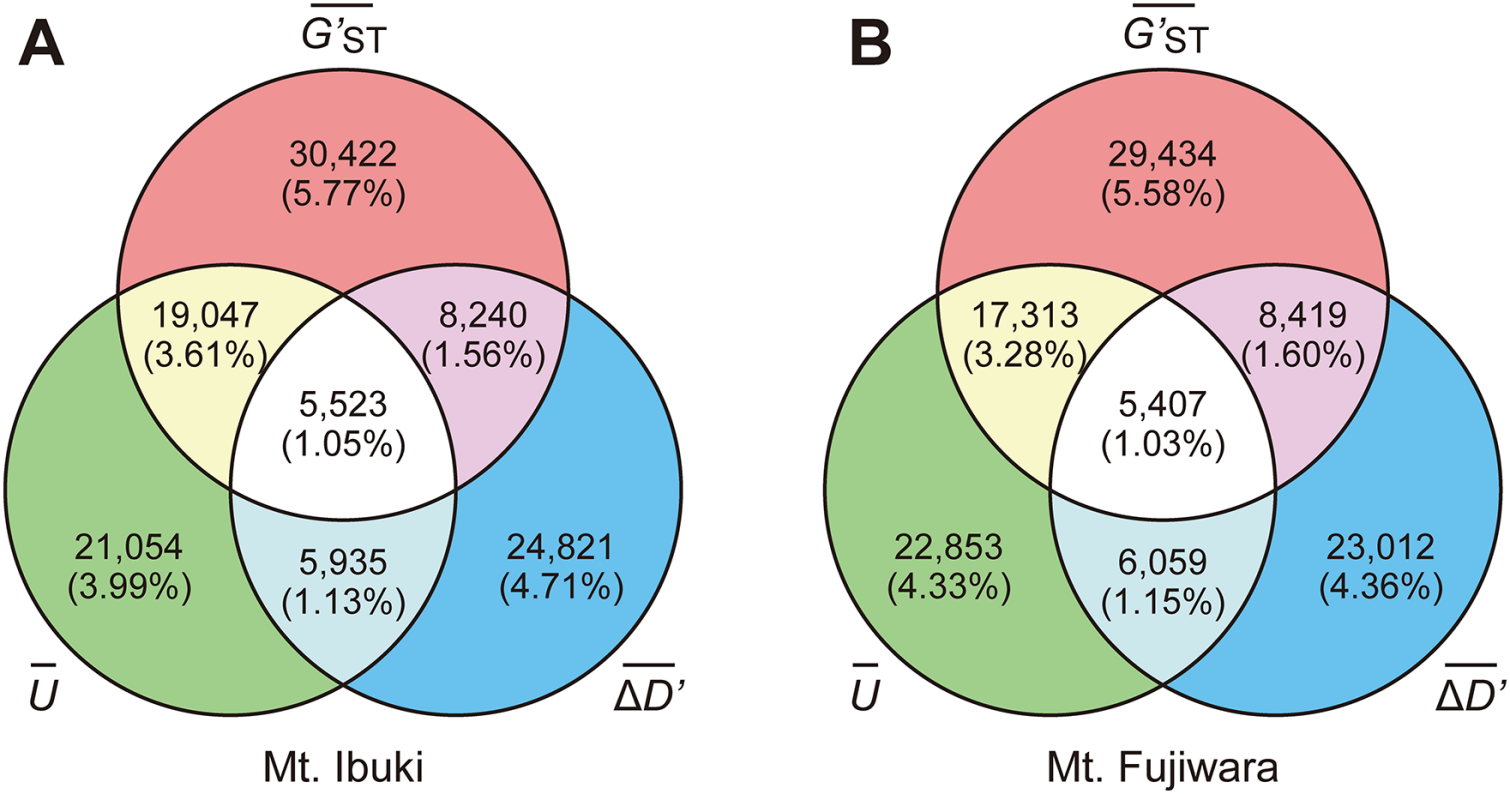
Overlap of screened SNPs among the three criteria. Venn diagram shows the overlaps of screened SNPs among the three criteria. The number of SNPs that fulfilled all three criteria were 5,523 (1.05% of the 527,225 SNPs) in Mt. Ibuki (A) and 5,407 (1.03% of the 527,225 SNPs) in Mt. Fujiwara (B).

To identify the SNPs associated with altitudinal adaption, we conducted a screening based on the following assumptions: first, and most importantly, we anticipated a cline in the allele frequency as a result of natural selection across environmental gradients. Therefore, we focused on those loci that undergo a unidirectional change in allele frequency along the altitudinal cline. To further reduce the number of candidate loci, we adopted the following two selection criteria: 1) the SNP loci should be highly divergent between the lowest and highest populations; and 2) the frequency of the derived allele should be higher in the highest-altitude populations. We developed an index *U* to measure the unidirectional change in allele frequency, used *G*′_*ST*_ proposed by Hedrick (2005 [39]) to measure the divergent between lowest and highest populations, and also developed an index Δ*D*′ to measure the frequency of the derived allele at the highest populations (see Materials and Methods section for details). Indices at each loci were averaged across a 4kbp window size and the upper 1.5 times the IQR (interquartile range) of a genome-wide frequency distribution (Fig 3) was determined as a screening threshold. Screening was conducted independently for the populations from each mountain, and only those SNP loci that fulfilled all three criteria were further considered. The number of SNPs that fulfilled the criteria was 5,523 for Mt. Ibuki and 5,407 for Mt. Fujiwara (Fig 4). The total number of identified SNPs in common between the two mountains were 358. Among the screened SNPs, 3,869 from Mt. Ibuki and 3,527 from Mt. Fujiwara were linked (overlapping or within 5 kbp of an exon) to a gene. The number of genes linked to the screened SNPs was 923 and 924 on Mt. Ibuki and Mt. Fujiwara, respectively.

**Fig 5:**
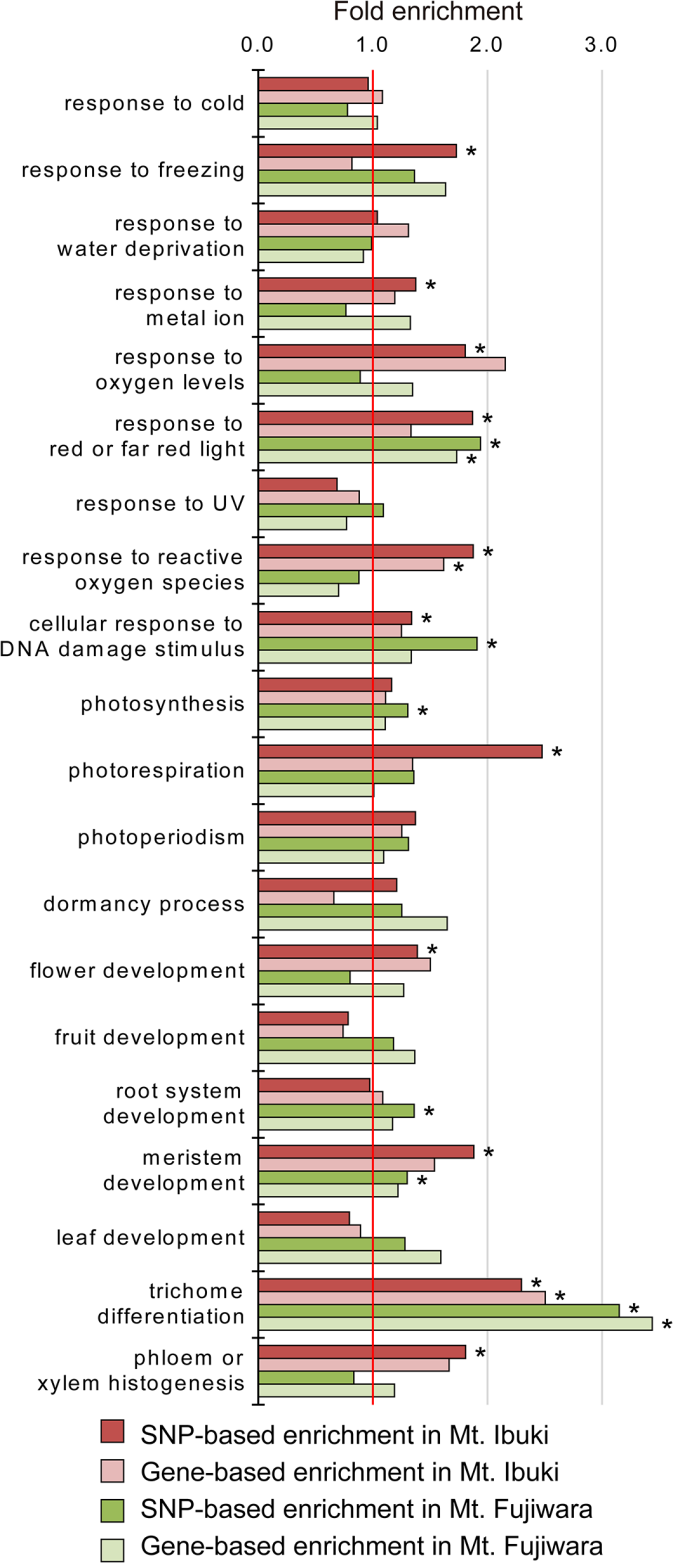
Enrichment analyses of the selected Gene Ontology terms. The histograms show the fold enrichment of a given GO term within each dataset for the two mountains. Vertical red line indicate the expected ratio of SNPs or genes associated with a specific GO term under the null hypothesis. Significant enrichment was accepted and denoted with asterisks if the corresponding false discovery rate (FDR) *q*-value was below 0.05. Here, we show only a subset of the tested GO terms. See S2 Table for the full list of GO terms.

To gain perspective into the biological process in which the screened SNPs are involved, we conducted a Gene Ontology (GO) enrichment analysis for each mountain. We tested for enrichment in 30 GO terms that cover the representative phenotypic and environmental entries within the database. To adjust for multiple comparisons, significant enrichment was accepted if the corresponding false discovery rate (FDR) *q*-value [40] was below 0.05. Here, we tested for enrichment using two approaches: one is an SNP-based method, where the ratio of SNPs that are associated and unassociated with a given GO term is compared between the lists of screened (SNP loci that fulfilled all three criteria mentioned above) and unscreened (all SNP loci) datasets. Another is a gene-based method, where the ratio of genes that are associated and unassociated with a given GO term is compared between the lists of screened and unscreened SNPs. Because the SNP-based method assumes that every screened SNP represents an independent observation, linkage between SNPs will cause bias, and the significance of enrichment will be overestimated [41]. However, the gene-based method ignores the joint effect of multiple SNPs within a gene, which may underestimate the significance of enrichment [41, 42]. As previously recommended for gene set enrichment analysis [43], we declare that our enrichment analysis is an exploratory procedure rather than a pure statistical solution. Not surprisingly, the SNP-based method detected more significant enrichment in GO terms compared with the gene-based method (Fig 5 and S2 Table). Here, we discuss the SNP-based enriched GO terms that were significant in both mountains. The four common GO terms were ‘response to red or far red light,’ ‘cellular response to DNA damage stimulus,’ ‘meristem development,’ and ‘trichome differentiation.’ It is noteworthy that the GO term related to trichomes, which constitute the most distinguishing characteristic of the highland ecotype [17], was detected in both mountains. In addition, enrichment for ‘trichome differentiation’ was also indicated by the gene-based method in both mountains. Detection of a major defining characteristic of the highland ecotype supports the validity of our screening procedure. Although the adaptive significance of the denser trichomes in the highland ecotypes remains unknown, our result strongly suggests that the trait has evolved under an common selective pressure between the two mountains. Another common GO term related to morphogenesis was ‘meristem development.’ This GO term can be related to the morphological differentiation where plants at the lower altitude are characterized by their tall and spindly appearance, and highland ecotypes by their dwarf-like appearance (S1 Fig).

Another common GO term ‘response to red or far red light’ is also interesting since previous observation has detected a positive correlation between canopy openness and altitude in both mountains [19]. Although we could not observe an enrichment in the term ‘photosynthesis,’ the increased investment to photosynthetic components in the higher altitudes in both mountains could be related to an adaptation against light environment variance. In this context, measurement based on cyclobutane pyrimidine dimer has demonstrated that opened canopy at higher altitudes induce increased UV induced DNA damage. At the same time, a correlation between altitude and UV tolerance via accumulation of UV absorbing compound was also detected in both mountains [19]. Although enrichment in the term ‘response to UV’ was not detected, we succeeded to find a significant enrichment in the term ‘cellular response to DNA damage stimulus’ in both mountains. These coincidence point out a possibility that light environment is an important selective pressure for the convergent evolution between the two mountains. On the other hand, although tolerance against freezing seems as an indispensable ability for high-altitude adaptation, previous observation detected an increased tolerance only from the highland ecotypes of Mt. Ibuki [19]. GO enrichment analysis were consistent with this result, where significant enrichment of the term ‘response to freezing’ was detected in Mt. Ibuki, but not in Mt. Fujiwara. Overall, consistency between the enriched GO terms and known features of the highland ecotypes suggests that our screening procedure provided a good estimate for the SNP loci associated with altitudinal adaptation.

### Application of BayeScan and LFMM

Here, we also tested other popular approaches to find the loci under selection. We used BayeScan [44–46] to find the *F*_*ST*_ outliers between the lowest and highest populations, and LFMM (Latent Factor Mixed Models [47]) to find the loci that correlate with the altitude. As shown in S3 Fig, these typical outlier tests did not fit very well with our dataset, especially in terms of detecting statistically significant outliers. More specific, at the significance level of a FDR *q*-value = 0.01, none of the loci from both mountains were detected by the BayeScan analysis. In Mt. Ibuki,–log10(*q*-value) of even those with the most highly differentiated loci (loci that are fixed for one allele in the lowest, and fixed for another in the highest population) reached a ceiling around 1.0. The problem seems to be caused by our sampling design, where small number of individuals were collected from limited geographical points. According to the manual for BayeScan, statistical power to detect the outliers will be limited when small sample size is used. On the other hand, LFMM analysis detected 1,530 outliers (FDR *q*-value < 0.01) in Mt. Ibuki, however, none were detected in Mt. Fujiwara. In LFMM, the background population structure is modelled from a chosen number of latent factors (*K*), which corresponds to the number of neutral genetic structure of the data. Underestimated value of *K* leads to liberal tests with false positives, whereas overestimated *K* leads to conservative tests with false negatives. Here, we used *K* = 2 as a number of latent factor in both mountains. From the structure analysis, a genetic split was detected in Mt. Ibuki and *K* = 2 was statistically supported (S2A and S2C Fig). However, in Mt. Fujiwara, clear differentiation (*K* = 2) was not supported (S2B and S2D Fig). Thus, *K* = 2 for Mt. Fujiwara may have been an overestimate, leading to a conservative test with false negatives. Although we can run the LFMM with *K* = 1, such run will not account for background population structures and will produce a plethora of false positives because a large set of loci is correlated with the altitude. Overall, because typical outlier analyses expect a set of numerous individuals from variable locations (environment) as an input, our dataset would not be suitable for these tests. Another problem may be the linear stepping-stone population structure detected in our study sites (Table 1), where not only the adaptive loci but also a large set of neutral loci can be correlated with the altitude. Under this condition, it would be difficult to determine the cutoffs to correct for the underlying population structure.

### Candidate genes for altitudinal adaptation

Based on the screened SNPs linked to genes, we attempted to narrow down and sort the candidate genes according to the likelihood of having undergone natural selection. Here, we assumed that the presence of genetic hitchhiking represented a footprint of a selective sweep [48]. However, we acknowledge that variation in mutation rates, non-uniform recombination rates, and chromosomal rearrangements can also lead to differentiated genomic regions and clusters of genes that contribute to local adaptation are more likely to diverge together regardless of selective sweeps [49]. To detect local signatures of genetic hitchhiking, we scanned for continuous allele frequency clines (the primary criterion for screening the SNPs) around the screened gene-linked SNPs. Through an independent scanning procedure within each mountain, we identified 474 and 629 continuous hitchhiking regions, or ‘genomic islands,’ which included 573 and 721 genes in the populations from Mt. Ibuki and Mt. Fujiwara, respectively (see S3 Table for the genes within top 100 genomic islands). To reduce the false positive detection from a single SNP locus, genomic islands that contained only one screened SNPs were rejected and total of 350 and 203 genes from Mt. Ibuki and Mt. Fujiwara, respectively, were excluded. Based on the length of the continuous hitchhiking region (i.e., the length of linkage disequilibrium) and the steepness of the allele frequency clines (i.e., the difference in allele frequencies between lowest and highest populations), the genomic islands were ranked according to how likely they were to have undergone a selective sweep (see S4 Fig for workflow). Linkage disequilibrium can be disrupted by recurrent mutations and recombination events during the evolutionary time course; a higher ranking indicates that the genomic region experienced stronger and/or more recent natural selection.

**Fig 6:**
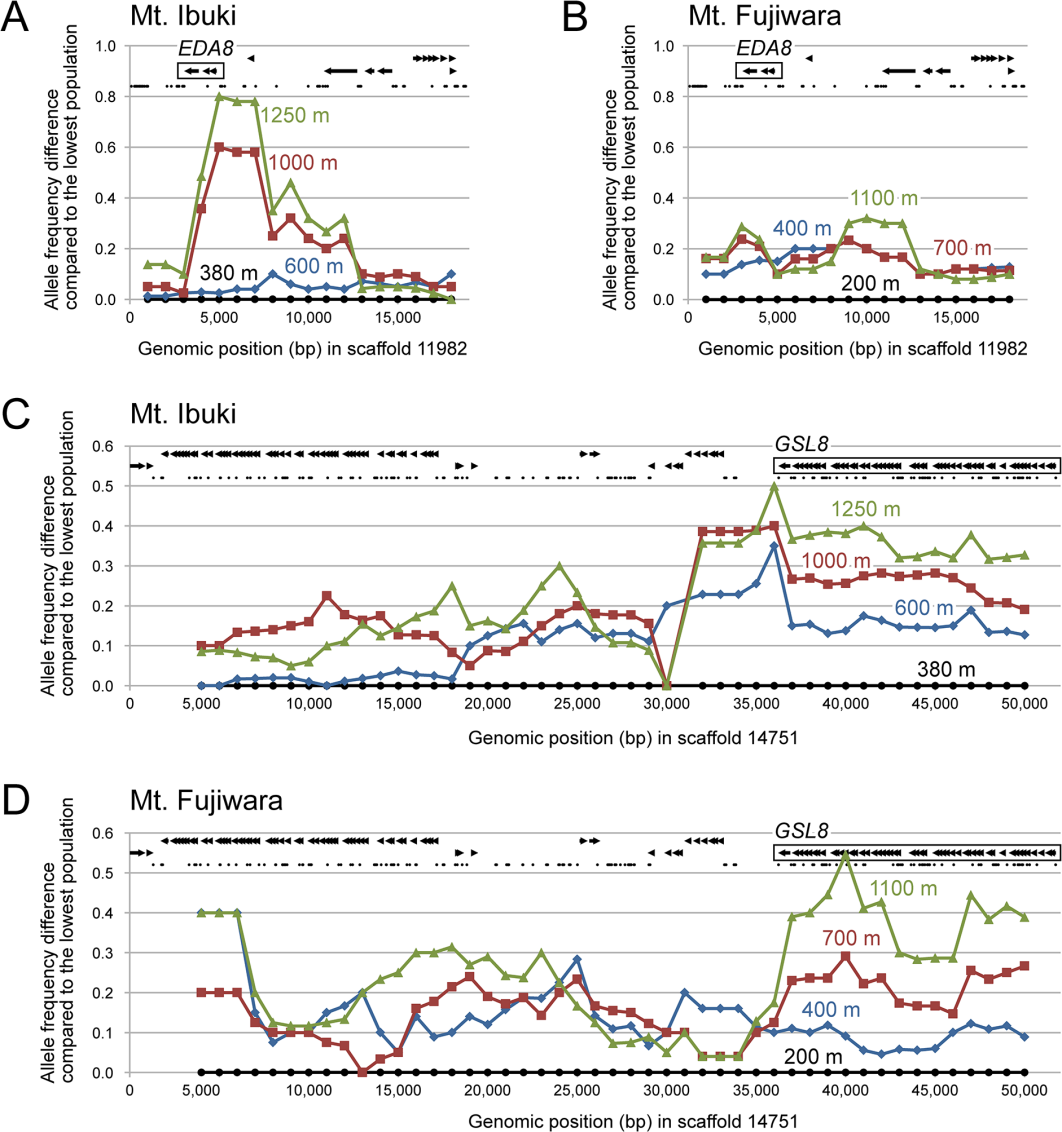
Local signature of a unidirectional allele frequency shift across altitudes. (A–D) The four colour-coded line graphs in each panel correspond to the allele frequency difference of the altitude-specific populations compared to the lowest population. Each dot of the line graph is an average of allele frequency differences 2 kbp down- and upstream from its genomic position (4 kbp window size). Arrows indicate the mapped exons of *A*. *thaliana* genes and small black dots represent the observed SNP positions. Continuous trend of unidirectional allele frequency shift was considered a footprint of natural selection and the proximal gene was accepted as candidates. (A, B) Example for a mountain-specific candidate gene. A steep allele frequency cline is found in 3 to 13 kbp regions of scaffold 11982 in Mt. Ibuki with a peak located near the 5’ UTR of *EDA8* (A). Conversely, no such trend is observed in the same genomic region in Mt. Fujiwara (B). (C, D) Example for a ‘shared’ candidate gene. Unidirectional allele frequency shift is detected from the 36 kbp and 50 kbp region (and most likely further) of scaffold 14751in both Mt. Ibuki (C) and Mt. Fujiwara (D). The region overlaps the exons of the *GSL8* gene. See Table 2 for other genes screened in our analysis.

Here, we considered the top 20 genomic islands as promising candidates that were recently subject to natural selection (Table 2). For example, we detected a steep allele frequency cline spanning approximately 10 kbp on Mt. Ibuki, with a peak near the 5’ UTR of *EDA8* (AT4G00310; Fig 6A). *EDA8* includes GO terms such as ‘regulation of flower development’, ‘response to freezing’, and ‘seed dormancy process’ [50]. Because freezing tolerance [19], flowering period, and seed dormancy (Shin-Ichi Morinaga, personal communications) differ between the lowest and highest populations from Mt. Ibuki, the functional annotations of *EDA8* are in line with the known phenotypic and environmental differences between altitudes. However, an allele frequency cline was not detected in the same genomic region on Mt. Fujiwara (Fig 6B). Mountain-specific candidate genes, such as *EDA8*, may indicate the underlying differences in natural selection between the mountains or that each mountain utilizes distinct genes to overcome a common natural selective pressure. Other genes from Mt. Ibuki with notable GO terms included the following: *FNR1* (AT5G66190), with ‘response to cold,’ and ‘photosynthesis’ [50]; *LIS* (AT2G41500), with ‘seed dormancy process,’ and ‘response to freezing’ [50]; *EMB2788* (AT4G27010) with ‘regulation of flower development’ [50]; *SAR1* (AT1G33410), with ‘regulation of flower development’ [50]; *FTSH12* (AT1G79560) with ‘embryo development ending in seed dormancy’ [51]; and AT5G16280 with ‘vegetative to reproductive phase transition of meristem’ [50]. Specific genes from Mt. Fujiwara included the following: AT2G40270 with ‘response to bacterium,’ and ‘response to insect’ [50]; *BAM7* (AT2G45880) with ‘vernalization response’ [50]; *STO* (AT1G06040), with ‘response to temperature stimulus,’ and ‘response to light stimulus’ [50, 52]; *AVP1* (AT1G15690), with ‘response to water deprivation,’ and ‘response to salt stress’ [53]; and *FWA* (AT4G25530), with ‘photoperiodism, flowering,’ and ‘trichome morphogenesis’ [50] (see Table 2). Detailed analysis of the adaptive roles of these mountain-specific genes in *A*. *halleri* subsp. *gemmifera* would highlight unique characteristics of natural selection in the superficially similar habitats between the two mountains. We also found that some genes within the list shared a common function. For instance, four genes from Mt. Ibuki (*EDA8*, *PBA1*, *FNR1*, and *LIS*) and three genes from Mt. Fujiwara (*PBA1*, *BAM7*, and *STO*) had GO terms under ‘response to temperature stimulus.’ Among the 22,395 SNP-tagged genes, only 863 were associated with this GO term, and an empirical *p*-value for the observed result was 0.007. Although increased freezing tolerance was detected only in highland ecotypes of Mt. Ibuki [19], our results suggest that temperature variation can be an important selective pressure for altitudinal adaptation in both mountains. Inferring environments and ecological traits from genomic information, the so-called ‘reverse ecology’ approach [54], may give rise to a new era in ecological genomics on wild plant species.

**Table 2:** Genes within the top 20 genomic islands from each mountain.

**Mt. Ibuki**			
**Rank**	**AGI code**	**Gene**	**Representative GO terms under ‘Biological Process’**
1	**AT1G67120**	*MDN1*	gluconeogenesis; cytoskeleton organization; embryo sac development
2	AT3G44713		
3	AT2G43160		
4*	**AT3G58160**	*XIJ*	actin filament-based movement; Golgi localization; mitochondrion localization
5*	AT4G00310	*EDA8*	seed dormancy process; leaf development; response to freezing
6	AT2G48060		
7* (2)	AT4G31300	*PBA1*	hyperosmotic response; response to temperature stimulus; response to cadmium ion
8	**AT4G34910**		protein import into nucleus
9*	AT1G80930		translation
10*	**AT5G66190**	*FNR1*	response to cold; detection of biotic stimulus; photosynthesis
11*	AT2G33820	*MBAC1*	mitochondrial transport
12*	AT4G04972		
13	AT1G28240		
14*	AT2G41500	*LIS*	meristem structural organization; seed dormancy process; response to freezing
15*	AT4G27010	*EMB2788*	embryo sac egg cell differentiation; regulation of flower development; maintenance of meristem identity
16	AT1G33410	*SAR1*	response to auxin; regulation of flower development; maintenance of meristem identity
17*	AT1G79560	*FTSH12*	chloroplast organization; embryo development ending in seed dormancy; ovule development
18 (1)	AT2G36850	*GSL8*	meristem initiation; trichome morphogenesis; telomere maintenance in response to DNA damage
19	**AT5G16280**		vegetative to reproductive phase transition of meristem; protein desumoylation; hydrogen peroxide biosynthetic process
20*	**AT4G32730**	*MYB3R1*	cytokinesis by cell plate formation; regulation of transcription, DNA-templated
**Mt. Fujiwara**			
**Rank**	**AGI code**	**Gene**	**Representative GO terms under ‘Biological Process’**
1 (18)	**AT2G36850**	*GSL8*	meristem initiation; trichome morphogenesis; telomere maintenance in response to DNA damage
2* (7)	AT4G31300	*PBA1*	hyperosmotic response; response to temperature stimulus; response to cadmium ion
3	AT2G41225		
4	AT4G30990		
5	AT2G40270		response to bacterium; response to insect; regulation of plant-type hypersensitive response
6	AT2G45880	*BAM7*	vernalization response; regulation of shoot system development
7*	AT5G63190		auxin-activated signaling pathway; response to sucrose; response to fructose
8*	AT1G06040	*STO*	hyperosmotic response; response to temperature stimulus; response to light stimulus
9	AT1G29400	*ML5*	fatty acid beta-oxidation; positive regulation of meiosis; positive regulation of growth
10*	AT3G03340	*UNE6*	positive regulation of cell proliferation; double fertilization forming a zygote and endosperm
11	AT1G63440	*HMA5*	response to zinc ion; detoxification of copper ion; response to copper ion
12*	AT2G38823		
13*	AT1G15690	*AVP1*	response to water deprivation; response to salt stress; leaf development
14*	AT1G25510		proteolysis
15	AT3G15300	*MVQ4*	
16*	**AT1G60780**	*HAP13*	intracellular protein transport
17	AT1G32750	*HAC13*	RNA splicing, via endonucleolytic cleavage and ligation; transcription from RNA polymerase II promoter; DNA mediated transformation
18	AT4G25530	*FWA*	trichome morphogenesis; photoperiodism, flowering; cell wall organization
19	AT1G52830	*IAA6*	de-etiolation; response to auxin
20	AT2G46430	*CNGC3*	

Numbers in parenthesis indicate rank in the other mountain. Asterisks indicate the presence of other genes that are located in the same genomic island. AGI codes in bold indicates genes with nonsynonymous SNPs that were highly differentiated (*G*’_ST_ > 0.4) between the lowest and highest populations. See S3 Table for the extended list.

The most novel findings of this study are candidate genes that are shared between the two mountains. In total, two genes were ranked within the top 20 genomic islands on both mountains. An empirical *p*-value to find two common genes between two independent gene lists from a set of 22,395 SNP-tagged genes was 0.001, which supports the presence of convergent evolution involving the same genes. Interestingly, both genes had functional annotations relevant to altitudinal adaptation. One of these ‘shared’ genes is *GSL8* (AT2G36850), which is annotated with the GO terms ‘meristem initiation,’ ‘trichome morphogenesis,’ and ‘telomere maintenance in response to DNA damage’ [50]. On both mountains, the genomic region around *GSL8* underwent a continuous unidirectional allele frequency shift that spanned at least approximately 15 kbp and most likely involved a longer region (Fig 6C and 6D). The long linkage distance observed in this case may be evidence of recent selection acting on this genomic region. In addition, anatomical observation of transposon-induced *gsl8 A*. *thaliana* mutant lines detected dwarfed growth, revealing the wild-type gene function in normal morphological development [55]. These results indicate that *GSL8* is an ideal candidate gene for explaining the morphological convergence found between the highland ecotypes on the two mountains. Another candidate is *PBA1* (AT4G31300), which presents the GO terms ‘response to temperature stimulus,’ ‘response to salt stress,’ and ‘response to cadmium ion’ [50, 56]. *PBA1* shows an altered expression level in response to various stresses, such as NaCl [56], zinc [57], genotoxic agents [58], oxidants [59], and viral infection [60]. Furthermore, an RNAi knockdown lineage showed defects in plant immunity against bacterial pathogens [61]. Considering the variety of functions related to abiotic and biotic stresses, *PBA1* appears to be a promising candidate for playing a role in altitudinal adaptation. Overall, these ‘shared’ genes may be a result of common natural selection acting on genetic variation that preceded the divergence of the two mountain populations, and they highlight the genetic basis of convergent evolution. Needless to say, other highly ranked genes without notable GO terms are also worth examining because they might retain unknown adaptive functions. To validate our result, the screened candidate genes must be further investigated by functional analyses of the genes, detecting loci that alter fitness, and field measurements including transplantation experiments.

Another ecological genomic study in *A*. *halleri* has been conducted at the Swiss Alps, where genome-wide SNP analyses were performed to search for the imprints from natural selection related to environmental variation [29]. By focusing on the highly differentiated genomic regions associated with environmental factors such as precipitation, slope, radiation, site water balance, and temperature, a list of 175 genes were obtained. Although the study case in the Swiss Alps was conducted in a wider geographical scale compared to the present study, the populations were situated at various altitudes ranging from 790 m to 2,308 m. Thus, we may have a chance to find common genes related to altitudinal adaptation between the mountains in central Japan (Mt. Ibuki and Mt. Fujiwara) and Swiss Alps. Unfortunately, none of the genes within the top 20 genomic islands from our study were found in the 175 genes from the Swiss Alps. However, three genes within each of the top 100 genomic islands from Mt. Ibuki and Mt. Fujiwara were also listed in the Alps (S3 Table). Although the coincidence is not surprising considering the large number of genes within each list (empirical *p*-value for the observed result was 0.09 for Mt. Ibuki and 0.06 for Mt. Fujiwara), we noticed that a single gene, *CMT1* (AT1G80740), was detected in all three locations (empirical *p*-value = 0.006). This gene was ranked as the 51st and 40th in the gene list from Mt. Ibuki and Mt. Fujiwara, respectively, and was associated with site water balance in the Swiss Alps. Although we must further compare the selected loci and haplotypes between central Japan and Swiss Alps, the gene may be an evidence of convergent evolution to altitude in different continents.

Although theories for local adaptation have supported the development of population genomics, several central predictions remain untested, especially for predictions involving gene flow (reviewed in [1]). Under gene flow, adaptive differentiation requires an allele with high fitness in one environment to show lower fitness in the other environment [62]. Thus, fitness trade-offs of the adaptive traits are expected to be associated with trade-off at the loci level. Otherwise the allele with the highest fitness will invade the other population thereby causing the locus to become monomorphic [63]. In addition, the loci involved in local adaptation are expected to cluster together on the chromosomes [14, 49, 64]. Further investigations on our candidate genes should provide an opportunity to empirically evaluate the untested predictions, and help understand the evolutionary dynamics of adaptive genes during local adaptation. In this context, an improved reference genome with longer scaffolds would not only enhance accuracy of detecting the selected genes, but also would assist in clarifying the positional relationship among the adaptive loci. The Joint Genome Institute (JGI) has recently assembled another reference genome for *A*. *halleri* which is available at: http://phytozome.jgi.doe.gov/pz/portal.html#!info?alias=Org_Ahalleri_er. Although dataset usage is restricted prior to publication, the reference genome from JGI has shorter total genome size (145.5 Mbp versus 252.2 Mbp) but longer N50 value (24.4 Kbp versus 4.8 Kbp), compared to our present reference genome. However, we are also developing an improved version of the *A*. *halleri* subsp. *gemmifera* reference genome, which should be comparable to the *A*. *halleri* genome from JGI.

Our study demonstrates that typical outlier-based approaches (BayeScan [44–46] and LFMM [47]) have limitation in screening for the selected loci at a microgeographic-scale. Due to recent colonization event, not only the selected loci, but also a large set of neutral loci can show patterns of variation where allele frequencies change along the environmental gradient. In such cases, the selected loci may not differ from the genomic mean sufficiently to be considered as an outlier. We therefore suggest that a genomic region-based approach (genomic islands in the present study) which aims to detect the genetic hitchhiking regions may be more successful, rather than approaches that treat each locus as independent. Another promising approach would be a comparison between parallel environmental gradients. A study in sessile oak investigated whether SNP variation of candidate genes reflect the clinal pattern of bud burst along altitudinal and latitudinal gradients [65]. By comparing the results in the two parallel gradients, a set of genes showing imprints of selection in both gradients were obtained, which can be considered as evidence for convergent evolution. In the present study, we also utilized two independent but parallel altitudinal clines, where phenotypic observations indicate the presence of a convergent evolution. Because the probability of occasionally detecting the same gene from parallel environmental gradients is very low, the common genes appear intuitively promising. We anticipate that the number of ecological genomic studies on convergent evolutions will grow, as it provides an excellent opportunity to efficiently screen the candidate genes responding to natural selection.

## Materials and Methods

### Study sites and sampling of materials

*Arabidopsis halleri* subsp. *gemmifera* is a perennial, self-incompatible, clonal herb distributed in the Russian Far East, northeastern China, Korea, Taiwan, and Japan [66]. The highland ecotype, characterized by denser trichomes, was formerly treated as the variant *Arabis gemmifera* var. *alpicola* [17] and is found only in the higher altitudes of Mt. Ibuki and Mt. Fujiwara in central Japan. On both mountains, continuous variation in morphological characters is found along altitudes (Shin-Ichi Morinaga, personal communications). Our main study populations were located on Mt. Ibuki (IB0380, IB0600, IB1000, and IB1250) and Mt. Fujiwara (FJ0200, FJ0400, FJ0700, and FJ1100). The low-altitude reference populations were situated at Minoo (MN0220), Inotani (IN0230), Itamuro (IT0520), and Okunikkawa (OK0370). See Fig 1A and 1B and S1 Table for the location and coordinates. Leaf samples were collected from each of the 12 populations and silica-dried for subsequent DNA extraction. To avoid sampling of clones, the sampled individuals were at least 4 m apart from each other.

### Establishment of the reference genome of *Arabidopsis halleri* subsp. *gemmifera*

Genomic DNA was extracted from the dried leaf of a single individual using the DNeasy Plant Kit (QIAGEN). This individual was collected from population IB0380 and was not included in the resequencing analysis. DNA libraries were prepared using the Illumina TruSeq DNA Sample Preparation Kit for paired-end reads, the Roche GS Titanium Rapid Library Preparation Kit for 454 single reads, and the SOLiD Mate-Paired Library Construction Kit for mate-pair reads. Instead of SOLiD adapters, Illumina adapters were used in the final step of mate-pair library construction. Reads were generated using the Illumina GAIIx, HiSeq2000 (300 bp paired-end reads, 3 kbp and 5 kbp mate-pair reads), and Roche 454 GS FLX Plus Titanium (single reads) systems. Subsequent data processing was performed with CLC Genomics Workbench version 6 (CLC bio). Raw reads were trimmed based on quality scores of 0.05 and a maximum allowance of two ambiguous nucleotides. Reads shorter than 60 bp for the Illumina platform and 100 bp for the Roche 454 platform were discarded. *De novo* assembly was carried out using the “De Novo Assembly” function with the following parameters: Mismatch cost 3, Insertion cost 3, Deletion cost 3, Length fraction 1, Similarity 1, Minimum contig length 200. Single reads from the Roche 454 platform were used as guidance-only reads. The number of reads used to construct the reference genome was as follows: 74,102,134 (7,034,411,911 nt) Illumina 300 bp paired-end reads, 150,099,682 (13,756,599,514 nt) Illumina 3 kbp mate-pair reads, 127,910,808 (11,644,031,026 nt) Illumina 5 kbp mate-pair reads, 66,195,930 (6,338,573,278 nt) single reads from the broken pairs of Illumina 3 kbp mate-pair reads, 73,840,719 (7,058,674,210 nt) single reads from the broken pairs of Illumina 5 kbp mate-pair reads, and 3,534,305 (2,579,555,709 nt) Roche 454 single reads. The established *de novo A*. *halleri* subsp. *gemmifera* reference genome sequences is uploaded online and freely available. The quality of the assembled reference genome was validated by mapping the exon sequence of *A*. *thaliana* at the TAIR10 database (The Arabidopsis Information Resource; http://www.arabidopsis.org). A total of 217,183 A. thaliana exon sequences were mapped using the “Map Reads to Reference” function with the following parameters: Mismatch cost 2, Insertion cost 2, Deletion cost 2, Length fraction 0.3, Similarity 0.9. Using the same parameter settings, the A. thaliana exon sequences were mapped to the reference genome of *A*. *lyrata* [33] downloaded from the JGI’s PHYTOZOME portal (US Department of Energy Joint Genome Institute; http://www.phytozome.net/alyrata).

### Individual-based resequencing, SNP discovery, and data cleaning

Genomic DNA from each of the 56 individuals was isolated with the DNeasy Plant Kit (QIAGEN). DNA libraries were constructed according to the Low-Throughput Protocol of the TruSeq DNA Sample Preparation Kit (Illumina). After quantification, 76 and 93 bp paired-end reads were obtained from the Illumina GAIIx platform and 101 bp paired-end reads from the HiSeq2000 platform. Raw short read sequences have been deposited at DDBJ and are freely available. Subsequent mapping and SNP calling procedures were performed using CLC Genomics Workbench version 6 (CLC bio). Prior to mapping, all sequences were trimmed based on a quality score of 0.05 and a maximum allowance of two ambiguous nucleotides. Broken pairs and reads shorter than 65 bp were discarded. For each individual, the reads were mapped to the reference genome with the following parameters: Mismatch cost 3, Insertion cost 3, Deletion cost 3, Length Fraction 0.97, and Similarity fraction 0.97. The reads from each individual were mapped to satisfy 9- to 15-fold coverage of the reference genome (S1 Table). We used 101 bp reads for mapping, but shorter reads were employed when the input was insufficient to meet the coverage demands. The short reads used for each individual are now undergoing the registration process and will be made freely available. SNPs were accepted if the locus had at least five reads per individual and the frequency of the antagonistic allele exceeded 20%. A total of 2 million provisional SNP loci were detected from the 56 individuals. Among these loci, those with a total read count over 10,000 were excluded because excessive read coverage may indicate nucleotide mismatches from paralogous copies of duplicated sequences. In addition, only those loci that had at least five reads in each individual were retained. Accordingly, a set of 527,225 SNP loci with an average read coverage per individual of 20 was obtained. Among these reliable SNP loci, 518,706 were bi-allelic, while 8,442 were tri-allelic, and 77 were tetra-allelic.

### Population structure analyses

A Bayesian clustering analysis of population structure was performed with structure version 2.3.4 [34, 35]. All 56 individuals from the 12 populations were subjected to analysis, and 10,000 SNP loci were randomly selected for the input dataset. Twenty independent runs for each value of *K* (the number of subpopulations) ranging from 1 to 12 were performed. For the optional setting for each run, we chose 400,000 iterations, with the first 200,000 iterations discarded as burn-in, and we applied the admixture model with correlated allele frequencies. To decide the best number of genetic clusters for the 56 individuals, we plotted the values of *LnP*(D) (log likelihood of the observed genotype distribution) and estimated Evanno’s Δ*K* [36] for each *K*. Based on the largest value of *LnP*(D) and a clear peak of Δ*K*, we selected 6 as the best *K* (Fig 2B). As we found further subdivisions within the mountains in runs with *K* above 6, we conducted additional analysis within each mountain. Using the same SNP loci and settings mentioned above, 20 individuals from each mountain were subjected to a set of analysis with *K* from 1 to 4. Although *LnP*(D) and Δ*K* supported *K* = 2 for Mt. Ibuki, genetic subdivision was not supported in Mt. Fujiwara (S2 Fig). Graphical representations of the results were generated using the program *Distruct* [67].

A maximum likelihood tree of the 12 populations was constructed with TreeMix version 1.12 [38]. This program uses a set of genome-wide allele frequency data from populations to construct the maximum-likelihood tree. Population splits are represented as nodes, and branch lengths are proportional to the amount of genetic drift experienced by the population. Migration events are inferred for populations that fit the tree poorly. Input allele frequency data for the 12 populations were generated based on 518,706 bi-allelic SNP loci. We first inferred the maximum likelihood tree without adopting a migration event, using OK0370 as an outgroup. To judge the confidence of the topology, 100 bootstrap replicates were performed. We then calculated the fraction of the variance in relatedness between populations that was explained by the tree (*f* of Equation 30 in [38]).

### Screening for SNPs associated with altitudinal adaptation

Screening of the 527,225 SNP loci was carried out according to the following three distinct criteria.

For the first criterion, we defined an index (*U*) to evaluate the level of unidirectional change in allele frequencies across altitudes. For each locus, the following index, ranging from −1 to 1, was calculated for each mountain:

$$U=|F_{L}-F_{H}|+\frac{\left|F_{L}-F_{H}|-|F_{L}-F_{M1}|-|F_{M1}-F_{M2}|-|F_{M2}-F_{H}\right|}{2}$$

where *F* indicates the allele frequency of a specific nucleotide in the lowest (*L*), lower-middle (*M*1), higher-middle (*M*2), and highest (*H*) altitude-specific populations. The nucleotide showing the largest allele frequency difference between the lowest and highest populations was used to calculate each *F*. The index yields greater values if the difference in the allele frequency between the lowest and highest populations is larger and if the allele frequency of the intervening population falls between that in the lower and higher populations. In other words, for a given allele frequency difference between the lowest and highest population, *U* value is highest when the frequency increases or decreases monotonically along the altitude. For each SNP locus, we calculated $\bar{U}$, which is an average of the *U* values 2 kbp down- and upstream (4 kbp window size) from its genomic position to minimize the spurious noise from single SNP locus.

The second criterion was used to evaluate the genetic difference of a given SNP locus between the lowest and highest populations within each mountain. For each SNP locus, Hedrick’s *G*′_*ST*_ [39] were calculated and averaged across 2 kbp down- and upstream from its genomic position to obtain $\bar{G\prime_{ST}}$.

Because the preceding two criteria basically filter those genes that are highly differentiated between lowest and highest populations, genes adaptive in the lower altitude can also be detected. While those genes are also interesting, our study system focus on high-altitude convergent evolution in two distinct mountains, and thus needed a third criteria to spot the genes that are related to high-altitude adaptation. Thus the third criterion was adopted to select those loci that show increased derived allele frequency (DAF) in the highest population compared with the low-altitude reference populations. Allele frequency data from the four reference populations (16 individuals in total) were combined and the allele with minor frequency was regarded as the derived allele. DAF of the reference populations ranges from 0 to 0.5, whereas DAF of the highest population ranges from 0 to 1.0. For tri- and tetra-allelic locus, we subtracted the major allele frequency from one and used it to calculate the DAF. An index to measure the increment of DAF in the highest populations (Δ*D*′) was calculated by:

$$\Delta D\prime =(|D_{H}-D_{R}|)(1-D_{R})$$

where *D*_*H*_ is the DAF in the highest population, and *D*_*R*_ the DAF in the reference populations. As we are not sure whether the allele is really ‘derived,’ especially for locus with high minor allele frequency in the reference populations, absolute value for the DAF difference is used. In addition, a probability of the allele being derivative (1 − *D*_*R*_) was used to correct the absolute DAF difference between the highest and reference populations. As well as other indices, Δ*D*′ values were also averaged 2 kbp down- and upstream (4 kbp window size) from its genomic position to obtain $\bar{\Delta D\prime }$.

For all three indices ($\bar{U}$, $\bar{G\prime_{ST}}$, and $\bar{\Delta D\prime }$), we analyzed the genome-wide frequency distribution and the upper 1.5 times the IQR of a genome-wide frequency distribution (Fig 3) was determined as a screening threshold. Screening was conducted independently for each mountain, and only those SNP loci that fulfilled all three criteria were considered further. Note that the three criteria are not completely independent. For instance, a steep monotonic allele shift along the altitude is likely to be found among loci that are highly differentiated between the lowest and highest populations. See Fig 4 for the overlaps between the sets of loci screened by different criteria.

### Gene Ontology enrichment analysis

To test for enrichment of a specific gene function among the screened SNPs, we conducted a Gene Ontology (GO) enrichment analysis with 30 GO terms that cover the representative phenotypic and environmental entries within the database. (See S2 Table for the complete list of the selected GO terms). Here, only those SNP loci that were linked (overlapping or within 5 kbp of an exon) to a mapped gene in the *A*. *halleri* subsp. *gemmifera* reference genome were used. The ratio between ‘the number of SNP loci (or genes) associated with a given GO term within the screened dataset’ and ‘the number of SNP loci (or genes) unassociated with a given GO term within the screened dataset’ was compared with the same ratio obtained from the unscreened dataset. Significant enrichment for each GO term was computed with a one-tailed Fisher’s exact test for a 2 × 2 table [68, 69], and *p*-values from multiple comparisons were adjusted using a 0.05 threshold of the FDR *q*-value [40].

### BayeScan and LFMM analysis

We also applied our datasets to two popular outlier detection methods that take account of the underlying population structures. Both analysis were independently conducted in each mountain. BayeScan uses a hierarchal Bayesian approach to detect outliers from the locus-specific *F*_*ST*_ distribution [44–46]. The program is based on a multinomial Dirichlet model that covers a wide range of realistic demographic scenarios. In addition, the program can be used with small number of samples with the risk of a low power, but with no particular risk of bias. We run our dataset with BayeScan 2.1 using the default parameter settings (20 pilot runs for 5,000 length, 50,000 burn in followed by additional 50,000 iteration with a thinning interval of 10). Posterior probabilities for each locus were calculated and corrected by the FDR method implemented in the program. Outliers were identified at the 1% significant levels of the FDR *q*-value.

Another method LFMM (Latent Factor Mixed Models) uses a hierarchal Bayesian mixed model to detect outliers from correlations between environmental and genetic variation [47]. At the same time, the program infers the background levels of population structure based on principal component analysis. Population structure is modelled from a chosen number of latent factors (*K*), which corresponds to the number of principal components to describe the neutral structure of the data. Underestimated value of *K* leads to liberal tests with false positives, whereas overestimated *K* leads to conservative tests with false negatives. Here, based on the results from structure analysis, we used *K* = 2 as a number of latent factor in each mountain. Population altitudes shown in S1 Table were used as the environmental data for each individual. Using the program lfmm in the LEA package version 1.0 (LEA: an R package for Landscape and Ecological Association studies; http://membres-timc.imag.fr/Olivier.Francois/LEA/index.htm), we conducted 20 runs with a burn in number of 5,000 and a total of 10,000 iterations. FDR *q*-value [40] was calculated for each locus based on the outputted *p*-values.

### Gene sorting according to the likelihood of having undergone natural selection

The sorting process of the candidate genes was based on the level of unidirectional change in allele frequencies across altitudes ($\bar{U}$ described above) and the effect of genetic hitchhiking. First, the $\bar{U}$ values of all SNP loci were plotted and connected with a line across genomic regions. Continuous regions with positive $\bar{U}$ values, starting and ending at the x-intercept, were considered to be hitchhiking regions (genomic island). In addition to the x-intercept, the genomic islands were terminated if the neighboring SNP loci were more than 4 kbp apart. We then defined the *x*-axis as the base and computed the area inside each genomic island. Genomic islands that contained at least two screened SNP loci were sorted from those with the largest area. The top 20 genomic islands contained 38 and 32 genes in the Mt. Ibuki and Mt. Fujiwara populations, respectively (see S3 Table). Finally, to visualize the unidirectional change in allele frequency, the difference in allele frequencies between the lowest and higher populations was plotted using a sliding window approach with window size of 4 kbp and a step size of 1 kbp (see S4 Fig for workflow).

We also carried out a simulation-based analysis to confirm the statistical significance of our results. To calculate the empirical *p*-value for obtaining two common genes from the two independent gene lists, we performed one million trials of randomly selecting 38 (number of candidate genes within the list for Mt. Ibuki) and 32 (number of candidate genes within the list for Mt. Fujiwara) genes from a set of 23,395 genes (total number of analyzed SNP-tagged genes). For each trial, we examined the number of shared genes between the two lists. Similarly, we calculated the empirical *p*-value for detecting three and four genes with the GO term ‘response to temperature stimulus’ in two gene lists. Again, we performed one million trials of randomly selecting 38 and 32 genes from a set of 23,395 genes. This time, however, 863 of the 23,395 genes were tagged with the GO term ‘response to temperature stimulus’ and we counted the number of genes with the GO term in the two derived gene lists.

## Supporting Information

S1 Fig Morphology of the normal and highland ecotypes from the two mountains. Each photograph displays the typical morphology of ecotypes found at the altitudes of 380 m (A) and 1,250 m　(B) on Mt. Ibuki and 200 m (C) and 1,100 m (D) on Mt. Fujiwara. The normal ecotypes are characterized by a tall, spindly, and glabrous appearance (A and C) and the highland ecotypes by a hairy dwarf-like appearance (B and D).
(TIF)

S2 Fig Additional structure analysis within each mountain. (A, B) structure analysis with a *K* of 2 to 4 using 20 individuals from Mt. Ibuki (A) and Mt. Fujiwara (B). The results for each *K* is based on the simulation that provided the best Ln*P*(D) value among 20 independent runs. (C, D) Plotting of the mean Ln*P*(D) values from the structure analysis (blue dots) and Evanno’s Δ*K* (red dots) in Mt. Ibuki (C) and Mt. Fujiwara (D). Error bars indicate the standard deviation of Ln*P*(D) values from the 20 independent runs.
(TIF)

S3 Fig Outlier tests by BayeScan and LFMM. Estimated ‒log10(*q*-value) from BayeScan and LFMM are plotted for each SNP locus in Mt. Ibuki (A) and Mt. Fujiwara (B).
(TIF)

S4 FigWorkflow for the sorting procedure of the candidate genes.(A) Index for the unidirectional allele frequency shift (*U*) for all SNP locus was calculated and plotted along the genome. (B) To minimize the spurious noise from single locus, *U* values were averaged across 2 kbp down- and upstream from the genomic position to obtain $\bar{U}$. (C) The $\bar{U}$ values of the SNP loci were connected with a line, and each continuous region with positive $\bar{U}$ values, starting and ending at the *x*-intercept or either end of a scaffold, was considered as a single hitchhiking region (genomic island). By defining the *x*-axis as the base, the area inside each genomic island was calculated. In the case shown in the figure, each colored area of (a), (b), and (c) are calculated. The area of each genomic islands were sorted from highest to lowest. Only those genomic islands that included at least two screened SNPs were retained, and genes overlapping or within 5 kbp of a screened SNP locus were considered as candidate genes. (D) Genomic islands with a larger area show longer and stronger trends of unidirectional allele frequency shifts.(TIF)

S1 Table Details of the individuals and populations included in the present study. (XLS)

S2 Table Details of the Gene Ontology enrichment analysis. (XLS)

S3 Table Genes within the top 100 genomic islands from each mountain. (XLS)
