# Correction: A Genome Scan for Genes Underlying Microgeographic-Scale Local Adaptation in a Wild *Arabidopsis* Species

**DOI:** 10.1371/journal.pgen.1005488

**Published:** 2015-09-22

**Authors:** Shosei Kubota, Takaya Iwasaki, Kousuke Hanada, Atsushi J. Nagano, Asao Fujiyama, Atsushi Toyoda, Sumio Sugano, Yutaka Suzuki, Kouki Hikosaka, Motomi Ito, Shin-Ichi Morinaga

The email address for the Corresponding Author Shosei Kubota is incorrect. The correct email address is: skubota44@gmail.com.
