# Supplementary figures and images for: A Genome Scan for Genes Underlying Microgeographic-Scale Local Adaptation in a Wild Arabidopsis Species
Source: PLoS Genet. 2015 Jul 14;11(7):e1005361. doi: 10.1371/journal.pgen.1005361 (PMC4501782; doi:10.1371/journal.pgen.1005361)

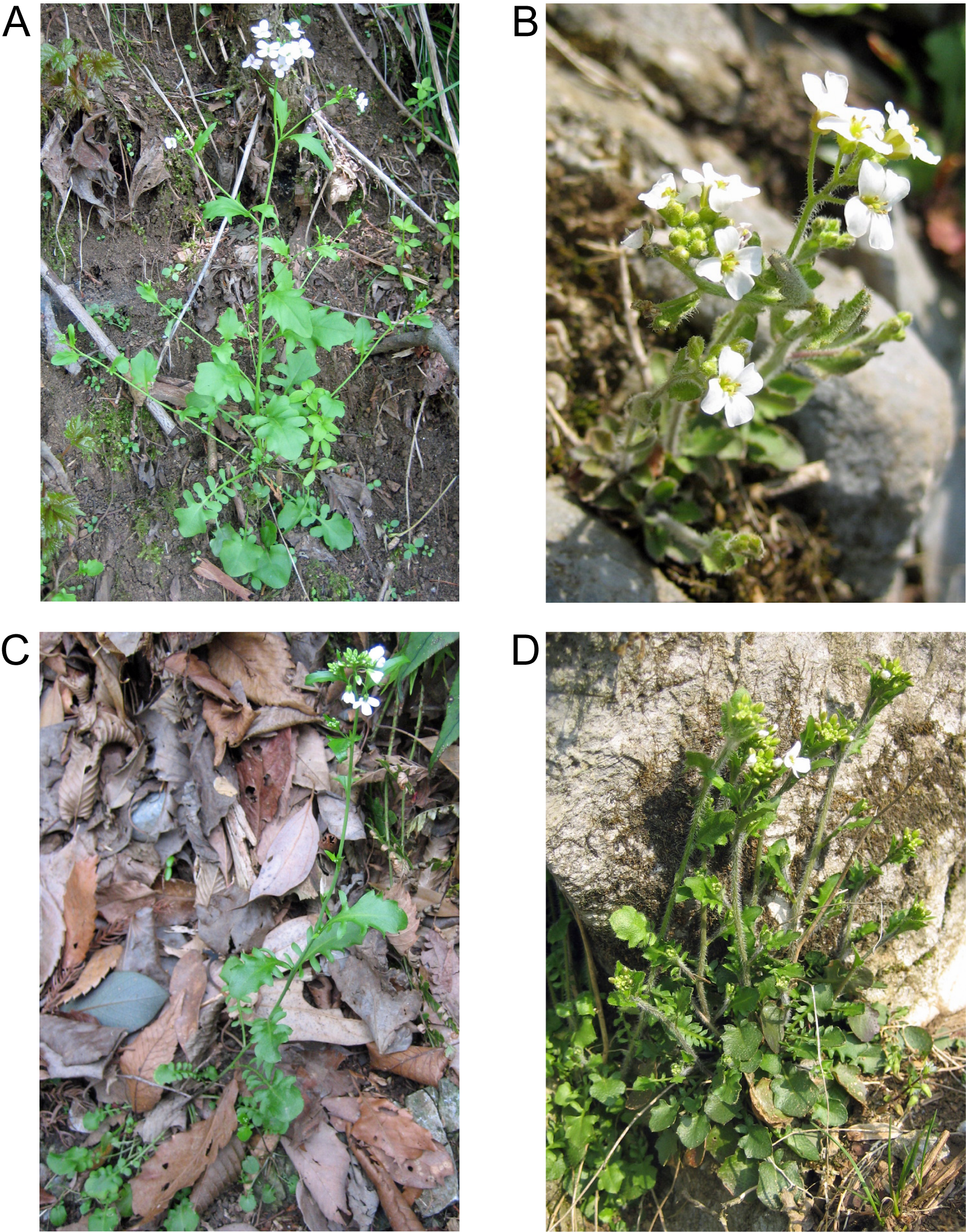

Supplement: S1 Fig — Each photograph displays the typical morphology of ecotypes found at the altitudes of 380 m (A) and 1,250 m (B) on Mt. Ibuki and 200 m (C) and 1,100 m (D) on Mt. Fujiwara. The normal ecotypes are characterized by a tall, spindly, and glabrous appearance (A and C) and the highland ecotypes by a hairy dwarf-like appearance (B and D). (TIF) [file pgen.1005361.s001.tif]

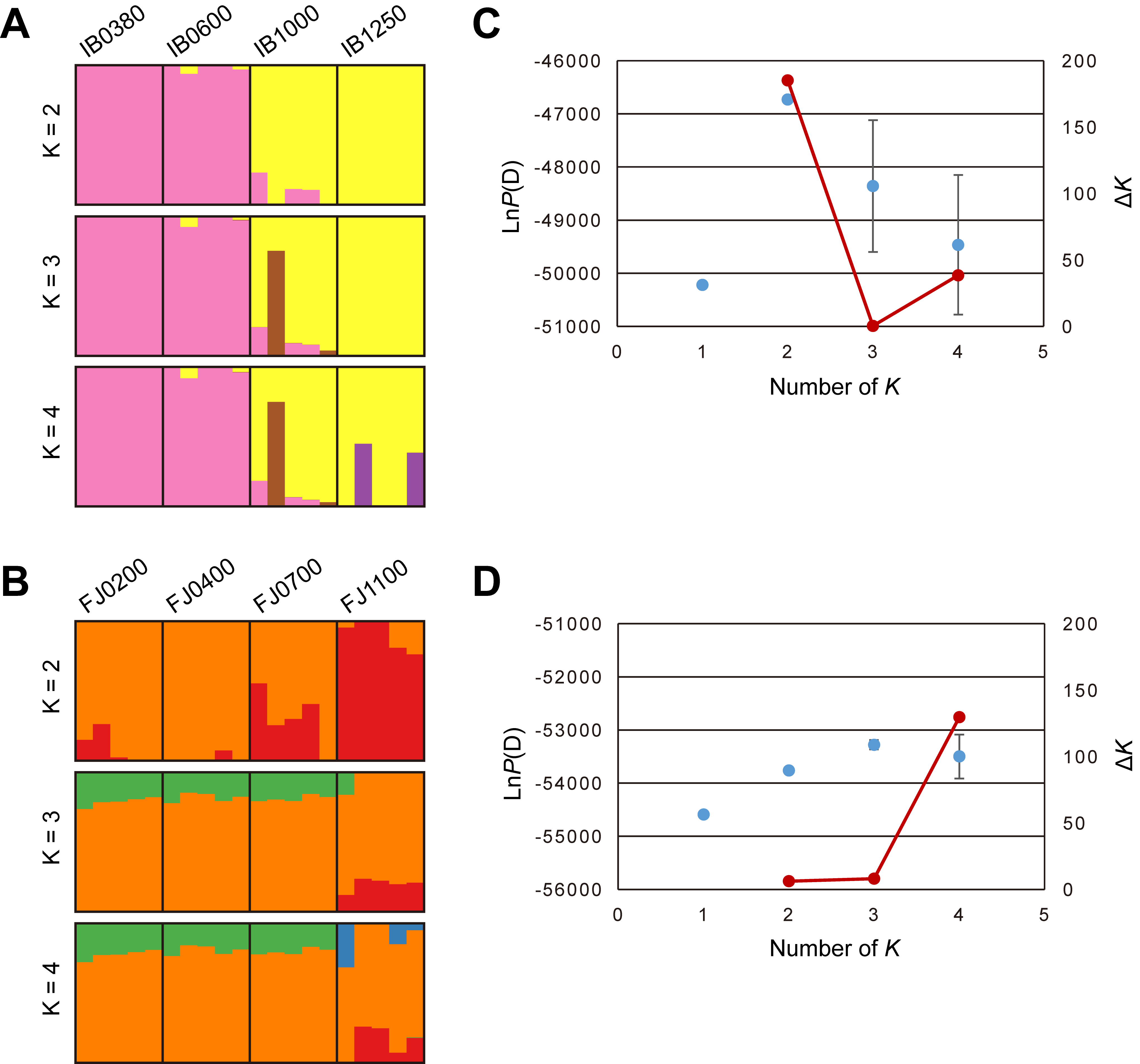

Supplement: S2 Fig — (A, B) structure analysis with a K of 2 to 4 using 20 individuals from Mt. Ibuki (A) and Mt. Fujiwara (B). The results for each K is based on the simulation that provided the best LnP(D) value among 20 independent runs. (C, D) Plotting of the mean LnP(D) values from the structure analysis (blue dots) and Evanno’s ΔK (red dots) in Mt. Ibuki (C) and Mt. Fujiwara (D). Error bars indicate the standard deviation of LnP(D) values from the 20 independent runs. (TIF) [file pgen.1005361.s002.tif]

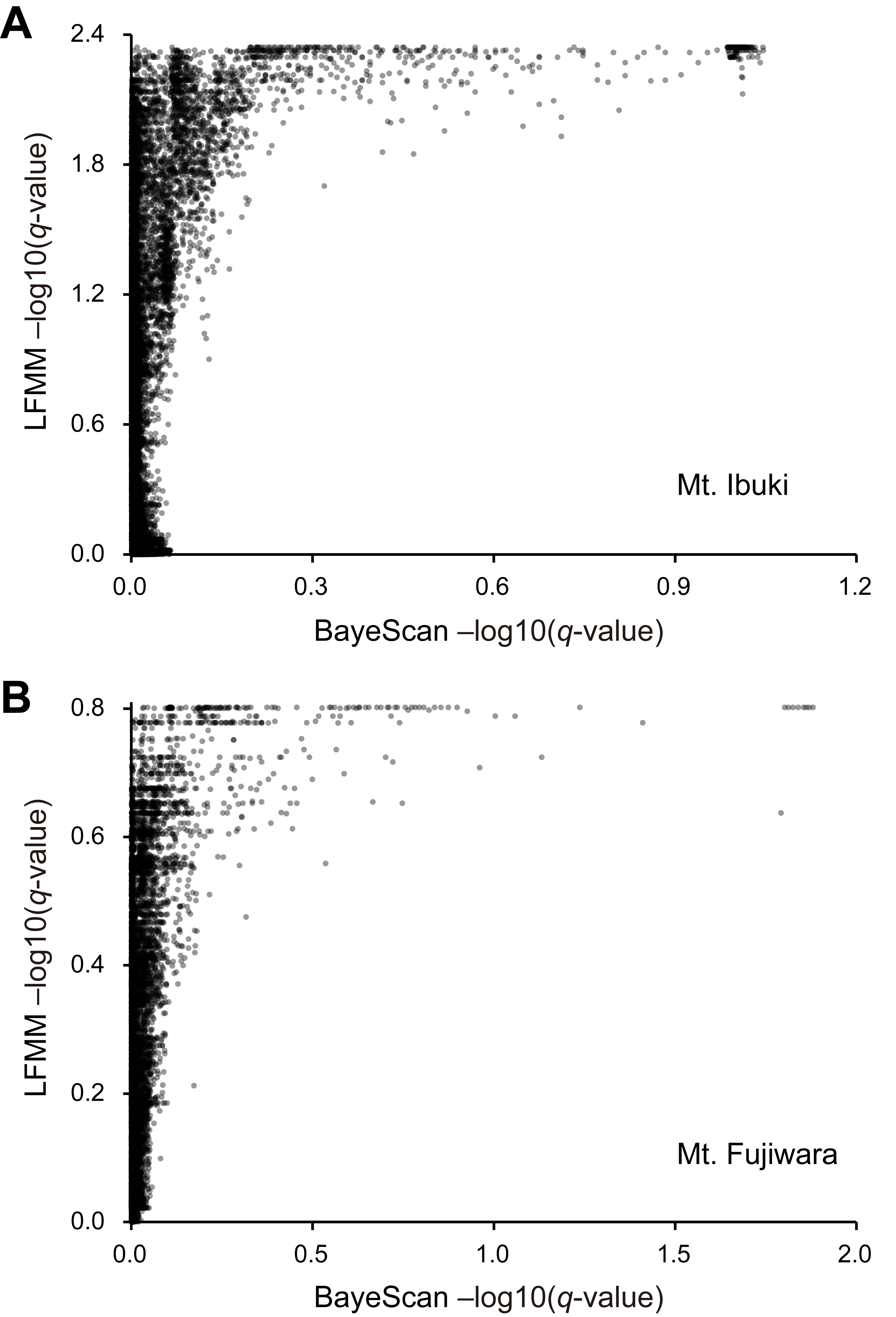

Supplement: S3 Fig — Estimated ‒log10(q-value) from BayeScan and LFMM are plotted for each SNP locus in Mt. Ibuki (A) and Mt. Fujiwara (B). (TIF) [file pgen.1005361.s003.tif]

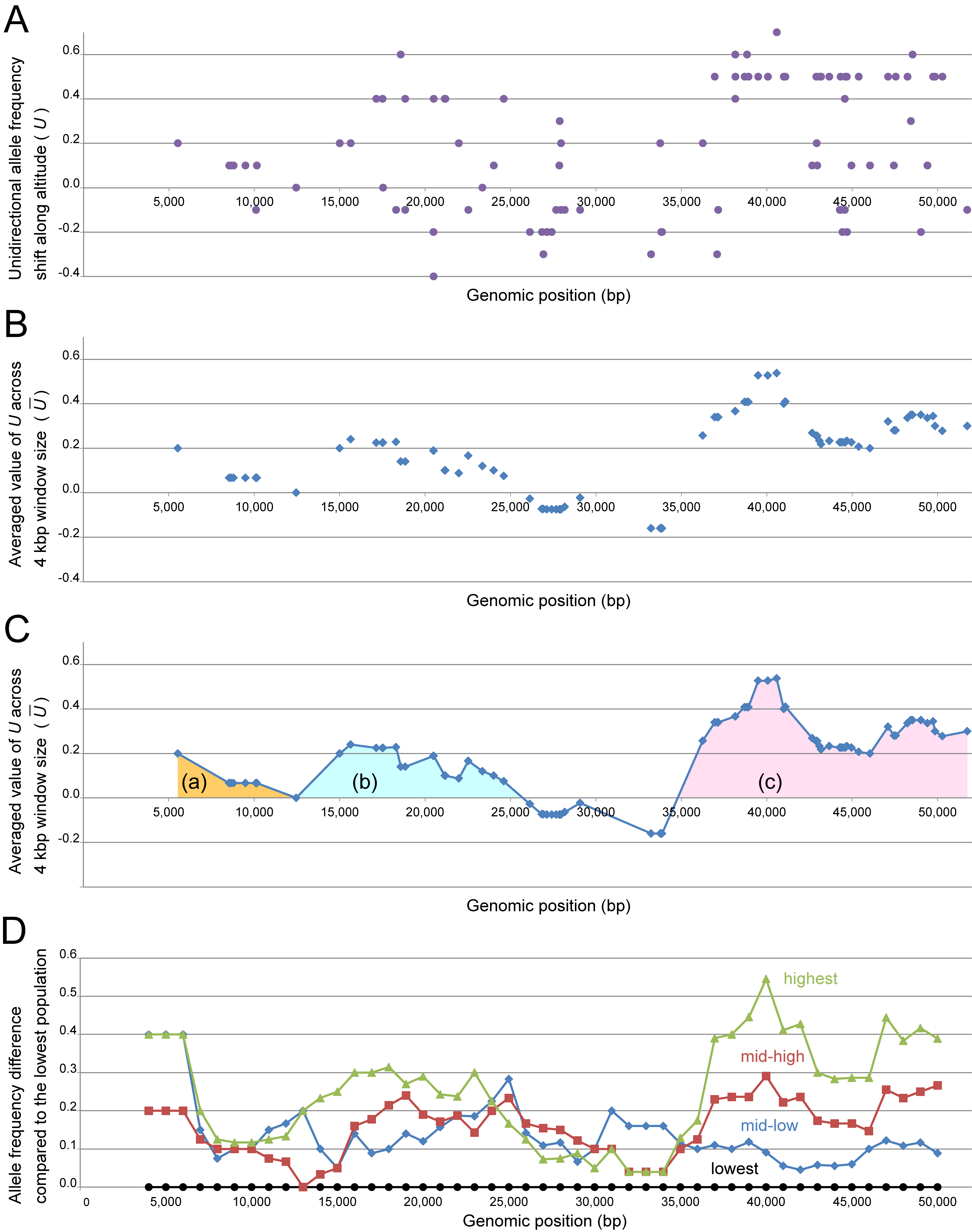

Supplement: S4 Fig — (A) Index for the unidirectional allele frequency shift (U) for all SNP locus was calculated and plotted along the genome. (B) To minimize the spurious noise from single locus, U values were averaged across 2 kbp down- and upstream from the genomic position to obtain U¯. (C) The U¯ values of the SNP loci were connected with a line, and each continuous region with positive U¯ values, starting and ending at the x-intercept or either end of a scaffold, was considered as a single hitchhiking region (genomic island). By defining the x-axis as the base, the area inside each genomic island was calculated. In the case shown in the figure, each colored area of (a), (b), and (c) are calculated. The area of each genomic islands were sorted from highest to lowest. Only those genomic islands that included at least two screened SNPs were retained, and genes overlapping or within 5 kbp of a screened SNP locus were considered as candidate genes. (D) Genomic islands with a larger area show longer and stronger trends of unidirectional allele frequency shifts. (TIF) [file pgen.1005361.s004.tif]
